# Aberrant cerebellar Purkinje cell function repaired in vivo by fusion with infiltrating bone marrow-derived cells

**DOI:** 10.1007/s00401-018-1833-z

**Published:** 2018-03-14

**Authors:** Kevin C. Kemp, Rimi Dey, Johan Verhagen, Neil J. Scolding, Maria M. Usowicz, Alastair Wilkins

**Affiliations:** 1 Multiple Sclerosis and Stem Cell Group, Translational Health Sciences, Bristol Medical School, University of Bristol, Bristol, UK; 2 School of Physiology, Pharmacology and Neuroscience, University of Bristol, Bristol, UK; 3 Infection and Immunity, School of Cellular and Molecular Medicine, University of Bristol, Bristol, UK; 4 Present Address: Department of Immunobiology, Faculty of Life Sciences and Medicine, King’s College London, Guy’s Hospital, London, UK

**Keywords:** Fusion, Purkinje cells, Cerebellum, Bone marrow-derived cells, Inflammation

## Abstract

Bone marrow-derived cells are known to infiltrate the adult brain and fuse with cerebellar Purkinje cells. Histological observations that such heterotypic cell fusion events are substantially more frequent following cerebellar injury suggest they could have a role in the protection of mature brain neurons. To date, the possibility that cell fusion can preserve or restore the structure and function of adult brain neurons has not been directly addressed; indeed, though frequently suggested, the possibility of benefit has always been rather speculative. Here we report, for the first time, that fusion of a bone marrow-derived cell with a neuron in vivo, in the mature brain, results in the formation of a spontaneously firing neuron. Notably, we also provide evidence supporting the concept that heterotypic cell fusion acts as a biological mechanism to repair pathological changes in Purkinje cell structure and electrophysiology. We induced chronic central nervous system inflammation in chimeric mice expressing bone marrow cells tagged with enhanced green fluorescent protein. Subsequent in-depth histological analysis revealed significant Purkinje cell injury. In addition, there was an increased incidence of cell fusion between bone marrow-derived cells and Purkinje cells, revealed as enhanced green fluorescent protein-expressing binucleate heterokaryons. These fused cells resembled healthy Purkinje cells in their morphology, soma size, ability to synthesize the neurotransmitter gamma-aminobutyric acid, and synaptic innervation from neighbouring cells. Extracellular recording of spontaneous firing ex vivo revealed a shift in the predominant mode of firing of non-fused Purkinje cells in the context of cerebellar inflammation. By contrast, the firing patterns of fused Purkinje cells were the same as in healthy control cerebellum, indicating that fusion of bone marrow-derived cells with Purkinje cells mitigated the effects of cell injury on electrical activity. Together, our histological and electrophysiological results provide novel fundamental insights into physiological processes by which nerve cells are protected in adult life.

**Electronic supplementary material:**

The online version of this article (10.1007/s00401-018-1833-z) contains supplementary material, which is available to authorized users.

## Introduction

Cells that reside within the bone marrow (BM) have long been known to fuse with several distinct types of cells throughout the body, including brain neurons [2, 41]. While the biological function of BM-derived cell fusion with neurons is not clear, related experiments involving fusion of ‘healthy’ donor BM-derived cells with host hepatocytes, to ameliorate metabolic liver disease, have provided strong clues for a role in targeted tissue regeneration [40, 43]. Within the brain, fusion of BM-derived cells occurs predominantly, although not exclusively [10], with Purkinje cells of the cerebellum [23]. Morphological and mRNA analyses of these fused Purkinje cells reveal the formation of either mononucleate cells or binucleate heterokaryons, with subsequent nuclear reprogramming and expression of donated nuclear genes [20, 29].

The incidence of BM-derived cells fusing with cerebellar Purkinje cells appears to be very low under normal physiological conditions [22, 24, 29]. Nevertheless, its biological relevance is suggested by observations that such fusion events in either rodents or humans are substantially increased in number with age [41, 42]; or after exposure to cytotoxic agents (for example radiation or chemotherapeutics) [26, 42]; or within an inflammatory microenvironment such as that present in multiple sclerosis [22] and in animal models of cerebellar disease [9, 10, 13, 20, 21, 29]. These observations have been taken to suggest that, as Purkinje cells are generated only during early cerebellar development [28], heterotypic cell fusion (fusion between different cell types) acts as a physiological cell rescue mechanism to counter neuronal injury and maintain Purkinje cell function throughout adulthood. Purkinje cells are some of the largest, most complex and elaborate neurons in the human brain; their axons represent the sole output for conveying nerve impulses from the cerebellar cortex, and as such, they are an essential part of the motor system [8]. Their loss is characterised clinically by insidious accumulation of disability [27]. If heterotypic cell fusion attenuates neuronal cell damage and prevents Purkinje cell dysfunction, it may have valuable therapeutic implications for neurodegenerative disease in general, and in particular, for patients with cerebellar injury.

The notion that fusion of BM-derived cells with adult Purkinje cells preserves or restores function in the face of injury currently remains an attractive, even likely, hypothesis. However, there is no direct experimental evidence of cellular repair; functional restoration in Purkinje cells has never been directly tested by assessing the cell morphology or electrical activity of the fused cells, or by comparing the properties of these cells with those of damaged Purkinje cells that have not undergone fusion with BM-derived cells or with Purkinje cells in healthy control animals. At a more fundamental level, it is not even known if an adult brain neuron that has fused with a different type of cell remains capable of firing action potentials; studying and elucidating these aspects of cell fusion in the adult brain is challenging. In an attempt to address these outstanding and crucial questions, we utilize an in vivo central nervous system (CNS) inflammatory disease model in chimeric mice expressing enhanced green fluorescent protein (EGFP)-BM cells. We perform both detailed histological and electrophysiological analysis of single Purkinje cells ex vivo in adult cerebellar slices, to assess the physiological role that cell fusion plays in neuronal protection in the adult brain.

## Materials and methods

### Animals

All animal experiments were performed in accordance with the UK Animals (Scientific Procedures) Act 1986 and approved by the University of Bristol Animal Welfare and Ethical Review Body. All animal numbers are based on both previous experience and/or power calculations using preliminary data. Blinding could not be performed when comparing Purkinje cells that had or had not fused with BM-derived cells due to reliance on EGFP to identify fused cells.

Transgenic mice ubiquitously expressing EGFP (strain # C57BL/6-Tg(CAG-EGFP)131Osb/LeySopJ, stock # 006567) were purchased from Jackson Laboratory, USA. Wild-type C57BL/6 VAF/Elite mice were provided by Charles River, UK. All mice were housed in a specific pathogen-free facility, with free access to food and water. If subject to a bone marrow transplant (BMT), mice were maintained in filter top cages (pore size 100 μm) during the recovery period.

### Bone marrow transplantation: generation of EGFP-expressing BM chimeric mice

Donor BM cells were harvested, under sterile conditions, from 10 to 12 week old male C57BL/6 EGFP-expressing transgenic mice [Tg(CAG-EGFP)131Osb/LeySopJ; Jackson laboratories, USA]. Briefly, mice were euthanized by cervical dislocation and BM cells harvested by gently flushing both their femurs and tibias (using a 27G needle) with phosphate buffered saline (PBS) (pH 7.4), 2% foetal bovine serum (FBS), 1% Penstrep, 10 units/ml heparin. Cells were subsequently passed through a 40 µm cell strainer, washed twice in PBS (pH 7.4) by centrifugation at 600*g* (10 min), and re-suspended in PBS to give a final concentration of ≥ 1 × 10^7^ cells/150 µl. Young adult female recipient wild-type C57BL/6 mice (aged 12 weeks) were irradiated, with a single dose of 1000 rad from a 137Cs source, 6 h prior to receiving 1 × 10^7^ unfractionated EGFP-expressing BM cells by tail-vein injection (there are well-established differences in the radiosensitivity of different inbred mouse strains [14]; C57BL/6 mice typically require a single dose of 900–1100 rad to achieve both complete myeloablation and high levels of BM engraftment). Sterile water, antibiotics (Baytril), sterile food and bedding were all provided for 4 weeks post-transplant.

### Detection of chimerism

At 12 weeks post BMT (female animals aged 24 weeks), haematopoietic reconstitution was evaluated in peripheral blood by flow cytometry (FACSCalibur, Becton–Dickinson). Briefly, 100 µl of peripheral blood was harvested from the tail vein and suspended in PBS [pH 7.4; ethylenediaminetetraacetic acid (EDTA), 2 mg/ml]. Red cells were removed using red cell lysis buffer, and the remaining nucleated cell population was re-suspended in PBS, 3% FBS and examined for EGFP expression when excited at 488 nm using flow cytometric analysis. Peripheral blood harvested from a non-transplanted C57BL/6 mouse was used as a reference control. Data were evaluated using BD Cellquest™ software.

### Induction and evaluation of experimental autoimmune encephalomyelitis (EAE)

At 18 weeks post BMT, mice (female, now aged ~ 30 weeks) were immunised by subcutaneous injection, at the base of the tail, of 100 µl of a sonicated emulsion containing equal volumes of complete Freund’s adjuvant (CFA) (Difco) and PBS containing 200 µg myelin oligodendroglial glycoprotein (MOG) peptide p35–55. CFA was supplemented with 4 mg/ml of heat-killed *Mycobacterium tuberculosis* (Difco). Pertussis toxin (Sigma Aldrich, P2980) (200 ng) was administered intraperitoneally in 500 µl of PBS directly after immunisation and again 48 h later. Individual mice were assessed twice daily for clinical signs of EAE using the following scoring system: 0, no disease; 1, flaccid tail; 2, hindlimb weakness and/or impaired righting; 3, hindlimb paralysis; 4, hind and forelimb paralysis; 5, moribund.

### Cerebellar slices

All female mice were culled aged between 9.5 and 11.5 months (~ 10 to 20 weeks after EAE induction), in accordance with the United Kingdom Animals (Scientific Procedures) Act 1986 and the University of Bristol Animal Welfare and Ethical Review Body. Parasagittal slices of cerebellar vermis (225 µm) were cut on a Leica VT1000S vibrating microtome (Leica Microsystems, Nussloch, Germany) in ice-cold solution (in mM: 62 NaCl, 124 sucrose, 1.3 MgSO_4_, 5 KCl, 1.2 KH_2_PO_4_, 26 NaHCO_3_, 10 D-glucose, 2.4, CaCl_2_, pH 7.4, bubbled with 95% O_2_, 5% CO_2_). They were stored in standard Krebs–Henseleit solution (in mM: 124 NaCl, 1.3 MgSO_4_, 5 KCl, 2.4 CaCl_2_, 1.2 KH_2_PO_4_, 26 NaHCO_3_, 10 d-glucose, pH 7.4, bubbled with 95% O_2_, 5% CO_2_) at room temperature for 1–8 h prior to extracellular recording from Purkinje cells. After recording, each cerebellar slice was fixed in 4% paraformaldehyde in PBS for 18 h at 4 °C and subsequently stored in PBS containing 0.1% sodium azide at 4 °C prior to immunohistochemistry. Cerebellar slices not used in extracellular recording were also fixed in 4% paraformaldehyde in PBS for 18 h at 4 °C and subsequently stored in PBS, 0.1% sodium azide at 4 °C. Slices were also made from female, age-matched control mice (9.5–11.5 months, C57BL/6 VAF/Elite).

### Immunohistochemistry and imaging

When required, cerebellar slices stored in PBS, 0.1% sodium azide were washed in PBS. For immunofluorescent labelling, non-specific binding was blocked with 10% normal goat/donkey serum diluted in PBS containing 0.1% triton. Sections were incubated at 4 °C overnight with primary antibodies to calbindin-D_28K_ (Sigma-Aldrich; C2724 1:500), CD11b/c (Abcam; ab1211 1:100), EGFP (Abcam; ab6556 1:500 and Abnova; MAB1765 1:1000), glutamic acid decarboxylase (GAD) (Abcam; ab11070 1:1000); myelin basic protein (MBP) (Serotec; MCA4095 1:100); synaptophysin (Dako; M7315), SMI-34 (Biolegend; 835503 1:500); zebrin II (kindly donated by Prof. Izumi Sugihara, Tokyo Medical and Dental University, Tokyo, Japan [39]). Sections were washed in PBS and incubated for 45 min in the dark with Alexa Fluor 488/555—goat/donkey anti-mouse (1:500),—goat/donkey anti-rabbit (1:500),—goat/donkey anti-rat (1:500) secondary antibodies (Invitrogen, Paisley, UK), and then mounted in Vectashield medium containing the nuclear dye 4′6′-diamidino-2-phenylindole (DAPI) (H-1200, Vector Laboratories).

A proportion of the cerebellar slices stored in PBS, 0.1% sodium azide were first embedded in paraffin for sectioning (8 µm) on a rotary microtome (Leica LM2135) and mounting on glass slides. Sections were deparaffinised in clearene, dehydrated in 100% ethanol and hydrated in distilled water. Antigen retrieval was performed, through boiling the sections in sodium citrate buffer (0.01 M, pH 6.0, 5 min), prior to immunofluorescent labelling (as described above). Confocal microscopic analysis was performed using either a Leica SP5-AOBS confocal laser-scanning microscope attached to a Leica DMI6000 inverted epifluorescence microscope or a Nikon C1 confocal microscope and EZ viewer software (Nikon). All Z-stack and three-dimensional imaging was created using Leica Application Suite Advanced Fluorescence software (Leica Biosystems) followed by Volocity 3D image software (PerkinElmer, USA).

### In situ hybridization

Cerebellar slices were embedded in paraffin for sectioning (8 µm) on a rotary microtome (Leica LM2135) and mounting on glass slides. Sections were deparaffinised in clearene, dehydrated in 100% ethanol, and placed in 0.2 M HCl for 20 min. The sections were washed in water and subsequently in 2× saline-sodium citrate (SSC) buffer before immersion in 10 mM citric acid (pH 6.0) at 80 °C for 2 h. After washing with water and then 2× SSC buffer, proteins were digested for 2 min at 37 °C using pepsin A (Digest-All 3 protease, Invitrogen). The sections were washed in water, dehydrated through an ethanol series and air dried. Fluorescent in situ hybridisation (FISH) probes (mouse control X and Y probes; Empire genomics, New York, USA) were prepared according to the manufacturer’s recommendations and applied directly to the tissue sections; a coverslip was subsequently placed on top and sealed using rubber cement. DNA was denatured at 83 °C for 5 min and then renatured with FISH probes by overnight incubation at 37 °C. The following day, slides were washed by immersion for 2 min in 0.4× SSC, 0.1% Tween-20 buffer at 73 °C, followed by 1 min in 2× SSC, 0.1% Tween-20 buffer, at room temperature. The sections were air dried and mounted in Vectashield medium containing the nuclear dye DAPI (H-1200, Vector Laboratories).

### Phenotypic analysis of fused Purkinje cells

Fused Purkinje cells in fixed, fluorescently labelled cerebellar slices were identified according to their location in the sagittal slice, characteristic morphology and co-expression of EGFP and calbindin-D_28K_. Each slice was scanned along the entire length of the Purkinje cell layer, situated between the granular layer and molecular layer. At least 2000 Purkinje cells from each mouse were examined to determine the frequency of Purkinje cell heterokaryons. The presence of two nuclei in these Purkinje cells was confirmed using confocal microscopy to view serial sections throughout the whole Purkinje cell soma. The frequencies of Purkinje cells expressing SMI-34 in the soma or with hypertrophic (enlarged) axons expressing SMI-34 were counted, scanning the entire Purkinje cell and granular layers.

FISH was also used to visualise nuclear chromosomal content of Purkinje cells. For X/Y chromosomal numeration and identification of Polyploid cells, FISH-labelled sections were viewed using confocal microscopy. Fluorescently labelled X and Y probes yielded green and red signals, respectively. Using confocal microscopy, cells were again scanned throughout the entire cell soma to observe the X/Y chromosomal frequency.

The cell soma area was measured in fixed sections stained with antibodies to EGFP and/or calbindin-D_28K_ by manually outlining the perimeter in Z-stacked confocal microscopic images using Image J software (National Institutes of Health). Measurements were made of EGFP-positive/calbindin-D_28K_ positive and adjacent or nearby EGFP-negative/calbindin-D_28K_ positive Purkinje cells (*n* = ≥ 5 animals, ≥ 10 cells measured/per animal). ≥ 16 individual cells/group were used to compare the soma size of EGFP-positive Purkinje cells containing two dispersed nuclei and EGFP-negative Purkinje cells with both a dispersed and a compact nucleus.

### Quantifying cerebellar inflammation and demyelination

CD11b/c was used to identify macrophages/microglia within tissue sections. All EGFP or CD11b/c positive cells were counted, using at least six randomly assigned fields per slice within both the white matter and grey matter regions of the cerebellum. The number of EGFP or CD11b/c positive cells per mm^2^ was subsequently calculated.

The degree of demyelination in both the cerebellar white and grey matter (granular layer) was assessed by quantifying anti-MBP labelling by optical density. Using at least three randomly assigned sagittal cerebellar slices per animal, confocal images of MBP immuno-labelled cerebellar sections from each animal were processed using Image J and MBP density was calculated as a percentage of the total area.

### Extracellular recording from Purkinje cells and analysis

Individual slices were viewed on a Zeiss FS Axioskop microscope (Carl Zeiss Ltd., Welwyn Garden City, UK) 1–8 h after cutting and superfused with standard Krebs–Henseleit solution at near physiological temperature (~ 34 ± 1 °C, maintained with a home-made Peltier-controlled recording chamber). Purkinje cells were readily identified by their position in the slice and characteristic size and shape. In slices from female mice with EAE, Purkinje cells fused with BM-derived cells were identified by EGFP fluorescence. Since < 1% of Purkinje cells were EGFP-positive, it was rare for such a cell to be at the surface of a slice and readily accessible for recording. Therefore, once an EGFP-positive cell was identified, it was often necessary to remove overlying tissue prior to recording. This was achieved with a fine stream of extracellular solution from a macropipette (tip diameter, ~ 5 µm). Extracellular recordings were made from the soma for at least 10 min in loose cell-attached mode with low resistance pipettes (1.2–1.9 MΩ, thin-walled borosilicate glass; GC150Tf-10, Harvard apparatus; filled with extracellular solution). To ensure that spontaneous firing was not influenced by the presence of the pipette on the soma, we prevented formation of a tight ‘seal’ between the pipette and soma by pre-exposing the pipette tip to the tissue in the slice [1]. The ‘seal’ resistance was < 9 MΩ. Spontaneous electrical activity was recorded in voltage-clamp with an Axopatch 200A or 200B amplifier (Axon Instruments, Union City, CA). Current recordings were low-pass filtered at 5 kHz (4 pole Bessel filter in the Axopatch 200 A/B amplifier) and digitised on-line at 40 kHz with a Cambridge Electronic Design (CED) power 1401 A/D interface using Spike2 software (v. 6.16) (CED, Cambridge, UK). In slices from mice with EAE, recordings were made from EGFP-positive cells and adjacent or nearby EGFP-negative cells. Recordings were also made from Purkinje cells in slices of sex- and age-matched control mice (female, no BMT, no EAE induction, 9.5–11.5 months).

Recordings were analysed with CED Spike2 software. Three different patterns of firing of control Purkinje cells were defined by the presence or absence of distinct, long pauses in firing (> 1.6 s), the distribution of interspike intervals (ISI) during firing periods and the shape of plots of instantaneous frequency (IF) against time. For tonically firing cells, the ISI distribution showed a single peak with a mean between 8 and 21 ms and a coefficient of variation (CV) between 0.08 and 0.80. This tonic category includes cells firing persistently in a regular tonic pattern (mean, 9–21 ms: CV, 0.08–0.21; e.g. see Online Resource 1; Fig. S1ai) or a regular tonic pattern interrupted by bursts of firing (mean, 8–13 ms; CV, 0.28–0.85; e.g. see Online Resource 1; Fig. S1aii). For cells firing in a trimodal manner (see Online Resource 1; Fig. S1b), there were distinct long pauses in firing (1.6–15 s) and the distribution of ISI during firing periods showed multiple components. The shortest ISI (< 10 ms) occurred within bursts and the longer ISI (25–50 ms) occurred between bursts, while intermediate ISI (~ 20 ms) separated spikes during regular tonic firing. The differing ISI resulted in a high CV (0.6–2.4). Cells were placed into this category if they showed all three modes of firing irrespective of the order; for example, it includes cells following a tonic, burst, pause, tonic, burst, pause pattern as well as cells following a tonic, burst, tonic, burst, pause pattern. For cells classified as firing in an irregular pattern (see Online Resource 1 Fig. S1c), spikes occurred as single events or in clusters of closely spaced two, three or four events. The ISI distribution was broad (2–500 ms) or it had two components, in which the first component represented intervals between spikes within clusters and the longer component represented intervals between clusters. The mean and CV ISI values were 15–43 and 0.8–1.3 ms. In some of these cells, the firing was separated by distinct pauses in firing (7–10 s).

### Statistical analysis

Statistical tests were performed using GraphPad Prism (v. 6 or 7, GraphPad Software Inc, USA). All tests were two-sided; values of *p* < 0.05 were considered statistically significant. For histological analysis, at least five independent animals from each group were included. Where data were known or predicted to violate assumptions for parametric statistical testing, an equivalent non-parametric test was performed (normality was analysed using the Shapiro–Wilk test; unequal variances between groups were subsequently analysed using either the Bartlett’s test or *F* test). Data between two groups were analysed using paired/unpaired Student’s *t* tests, Wilcoxon matched-pairs signed rank tests or Mann–Whitney *U* tests. Statistical comparisons for more than two groups were analysed using either one-way analysis of variance (ANOVA) followed by Holm–Sidak’s multiple comparisons test or Kruskal–Wallis followed by Dunn’s multiple comparison test between groups. Box and whisker plots represent the median, upper quartile and lower quartile values (box) and the minimum and maximum values (whiskers). For electrophysiological data, the Chi-squared test was used to compare the frequency of different modes of firing. One-way or two-way ANOVA followed by the Holm–Sidak’s multiple comparisons test was used to compare different firing parameters. Number of cells is denoted as *n*. Origin (v. 6 or 7, Microcal, Northampton, MA, USA) was used to plot voltage traces.

## Results

### Bone marrow transplantation leads to robust levels of BM chimerism

**Fig. 1:**
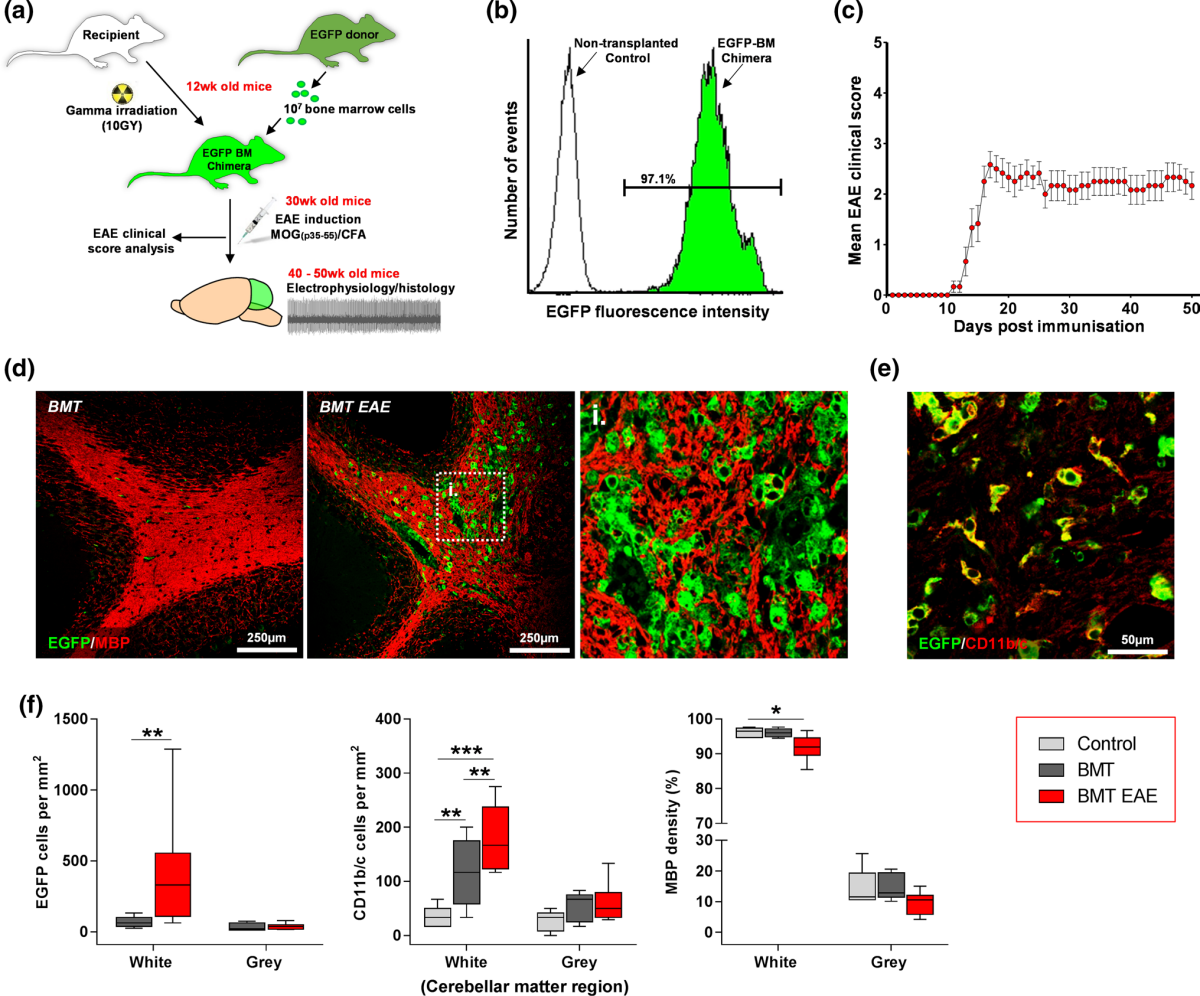
EGFP-BM chimera formation, EAE induction and detection of inflammatory demyelinating lesions in the cerebellar white matter. **a** A schematic representation of the experimental design. To form EGFP-expressing BM chimeras, recipient mice underwent myeloablative irradiation prior to receiving unfractionated BM cells expressing EGFP by tail-vein injection. Mice subsequently underwent EAE induction, with MOG (p35-55), and the clinical EAE score was monitored daily until animals were euthanised for electrophysiology and histological analysis. **b** Flow cytometric analysis to evaluate the level of BM chimerism within mice transplanted with EGFP-BM cells. The percentage of peripheral blood mononuclear cells (MNCs) positive for EGFP (green peak) was calculated [MNCs with a relative fluorescence higher than that of MNCs derived from non-transplanted wild-type control mice (white peak)]. **c** The mean clinical score of mice with EAE post immunisation. (0, no disease; 1, flaccid tail; 2, hindlimb weakness and/or impaired righting; 3, hindlimb paralysis; 4, hind and forelimb paralysis; 5, moribund). **d** Representative images of cells positive for EGFP in cerebellum labelled for MBP of BM-transplanted mice (BMT) and BM-transplanted mice with EAE (BMT EAE). After EAE, there are more EGFP-positive cells, EGFP-positive macrophages detected containing myelin debris within their cytoplasm, and patchy loss of MBP expression (demyelinating lesions; unlabelled patches). The hatched area in BMT EAE refers to the magnified image (i). **e** An enlarged image showing that EGFP-positive cells co-express the microglial/macrophage marker CD11b/c in the cerebellum of BMT EAE mice. **f** Quantification of EGFP-positive cells, CD11b/c-positive cells and MBP density within the cerebellar white and grey matter in control, BMT and BMT EAE mice. **p < 0.01 *EGFP cells* (**f**); Mann–Whitney *U* test. **p < 0.01, ***p < 0.001 *CD11b/c cells* (**f**); one-way ANOVA followed by Holm–Sidak’s multiple comparison test. *p < 0.05 *MBP levels* (**f**); Kruskal–Wallis followed by Dunn’s multiple comparison test. Box and whisker plots show median ± quartiles (box), min/max (whiskers). For all tests, *n* = ≥ 5 per group

To enable tracking of BM-derived cells infiltrating the brain, we generated BM chimeras by lethally irradiating wild-type C57BL/6 mice (to deplete their own BM) and stably reconstituting these animals with BM-derived cells expressing EGFP (donated by EGFP-expressing C57BL/6 mice, see methods) (Fig. 1a; abbreviated in the figures as BMT). All chimeric mice showed robust levels of chimerism; 97.1% ± 2.57 (mean ± SD, *n* = 8) of mononuclear cells within the peripheral blood of transplanted mice were EGFP-positive and thus donor-derived (Fig. 1b).

### Induction of EAE results in significant cerebellar injury

To induce cerebellar injury, inflammation of the CNS was triggered with EAE [38]; a widely studied animal model of human CNS inflammatory demyelinating diseases, including multiple sclerosis. Chimeric mice (*n* = 15) were immunised 18 weeks post-transplantation by subcutaneous injection of MOG peptide in adjuvant. Following EAE induction, the disease was observed to manifest clinically from approximately 10 days post-immunisation, with paralysis proceeding in a caudal to rostral pattern (beginning with the tail and progressing to the hind limbs) and stabilizing at day 17 (Fig. 1c). In several animals, an additional lack of coordination was evident, suggesting cerebellar involvement.

**Fig. 2:**
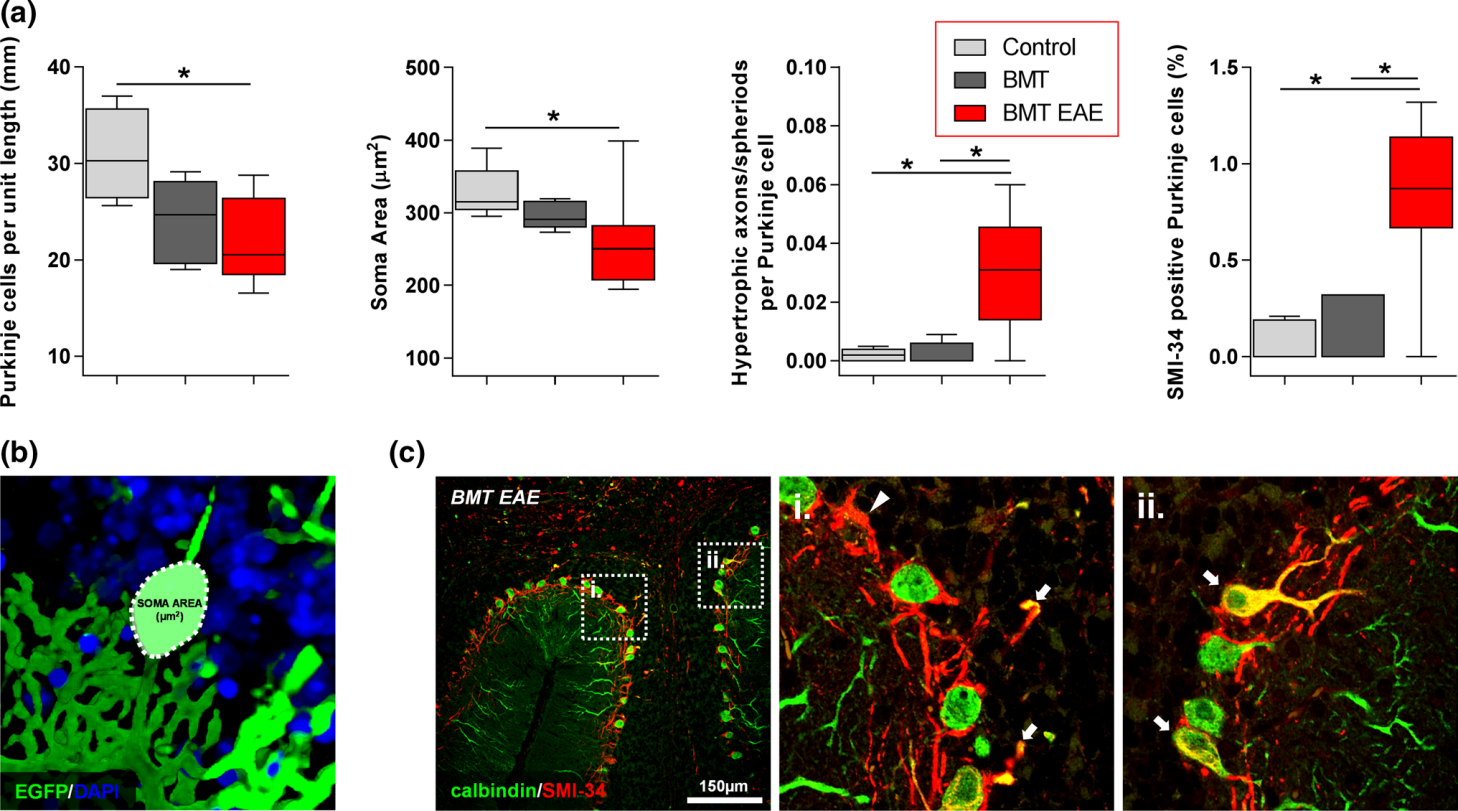
EAE induces Purkinje cell pathology in BM transplanted mice. **a** Quantification of Purkinje cells per mm of the Purkinje cell layer, size of Purkinje cell soma, the number of hypertrophic axons or axons with spheroid (normalised by Purkinje cell number) and the number of SMI-34 positive Purkinje cells in control mice, mice transplanted with BM cells (BMT) and mice transplanted with BM cells and EAE (BMT EAE). EAE decreased Purkinje cell number and soma size and induced cell injury as indicated by the increase in number of hypertrophic axons and cells expressing SMI-34. **b** A 3D laser-scanning confocal image depicting calculation of Purkinje cell soma area. **c** Injured Purkinje cells depicted through (i) Purkinje cell loss (empty basket; white triangle) and hypertrophic axons (white arrows) and (ii) SMI-34 positive Purkinje cells (white arrows) within the cerebellum of a BM transplanted mouse with EAE. Statistical comparisons are either one-way ANOVA followed by Holm–Sidak’s multiple comparison test [Purkinje cells per mm (**a**)] or Kruskal–Wallis followed by Dunn’s multiple comparison test [soma area; hypertrophic axons/spheroids; SMI-34 positive Purkinje cells (**a**)]. *p < 0.05. Box and whisker plots show median ± quartiles (box), min/max (whiskers). For all tests, *n* = ≥ 5 per group

Pathologically, ‘classical’ MOG-induced EAE in mice is associated with extravasation of activated myelin-specific T-lymphocytes into the CNS and subsequent recruitment of macrophages, which cause focal plaques of demyelination associated with both axonal damage and neuronal loss [30, 33]. In confirmation, we observed cerebellar white matter loss with cavitation and fragmentation of myelin, as indicated by a fall in the density of MBP, in the brains of mice with EAE from 10 weeks post-immunisation. These changes were accompanied by extensive infiltration into the cerebellum of activated EGFP-positive macrophage/microglial cells, which were found to contain cytoplasmic myelin debris (Fig. 1d–f). We observed a decrease in the number of Purkinje cells (identified by expression of calbindin-D_28K_ [31]) and a significant reduction in soma size (Fig. 2a, b), indicating Purkinje cell atrophy [24]. (These changes were likely caused not only by CNS inflammation, but also by gamma irradiation-induced injury during myeloablative BMT conditioning [44]). There were also rare instances of multifocal loss of Purkinje cells (absence of calbindin-D_28K_ labelling) with basket cell pinceau remaining in situ, resulting in the formation of ‘empty baskets’ (the axon terminals from basket cells that typically envelop Purkinje cell soma, appear to surround an empty space) (Fig. 2c). In addition, EAE caused Purkinje cell axonal thickening and formation of axonal torpedoes within the granular layer, indicative of injury to the axonal compartment [24] (Fig. 2a, c). Dual-immunolabelling with calbindin-D_28K_ and SMI-34 (anti-phosphorylated neurofilament) revealed pathological SMI-34 accumulation within the Purkinje cell soma, axon and dendrites, indicative of abnormally high levels of neurofilament phosphorylation [34] (Fig. 2a, c).

### Purkinje cells fused with BM-derived cells in EAE animals form binucleate heterokaryons that are otherwise morphologically typical of control Purkinje cells

**Fig. 3:**
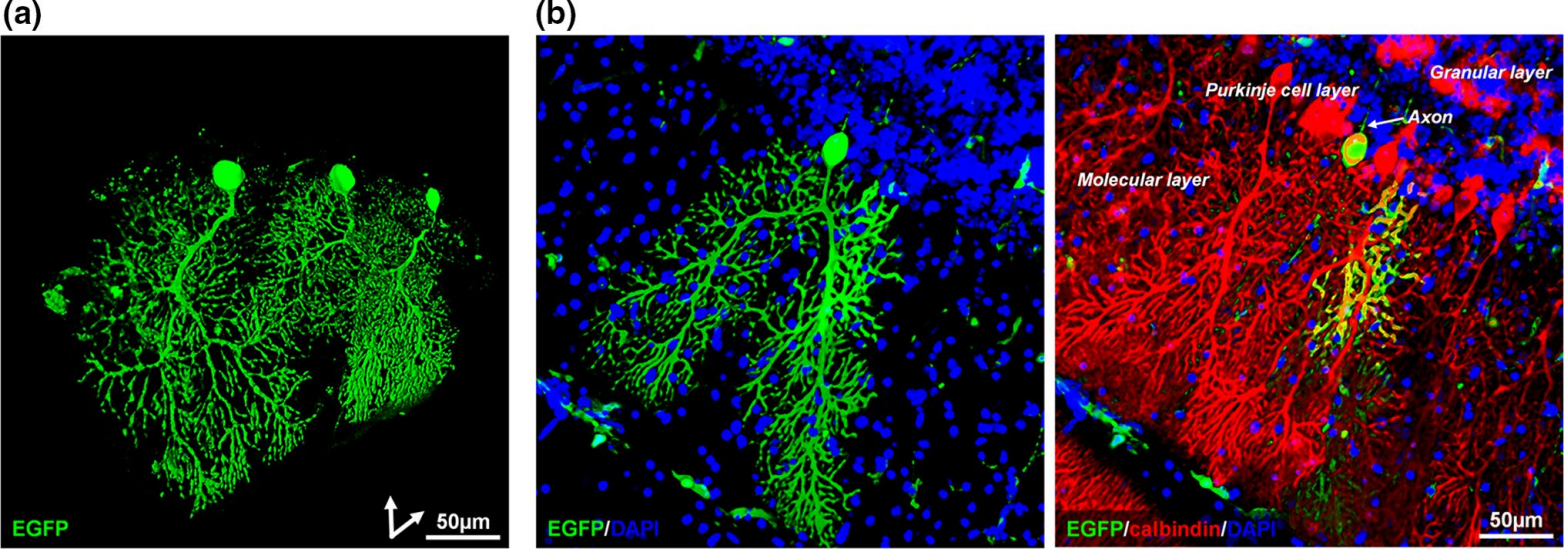
The shape of Purkinje cells fused with BM-derived cells in EAE animals is typical of control Purkinje cells. 3D laser-scanning confocal images of EGFP-positive Purkinje cells (cells that had fused with EGFP-expressing BM-derived cells) within the cerebellum of BM transplanted EAE mice. Examples of cells labelled for **a** EGFP or **b** EGFP plus the fluorescent nuclear stain DAPI, with (right) or without (left) the Purkinje cell-specific marker calbindin-D_28K_. Double immunolabelling revealed coexpression of EGFP throughout the soma, dendrites and axon in a Purkinje cell (green), amongst Purkinje cells positive only for calbindin-D_28K_ (red). EGFP-positive Purkinje cells showed typical Purkinje cell morphology; extensive dendritic arborisation climbing through the molecular layer toward the surface of the cortex and an axon originating at the opposite (basal) pole of the cell soma (within the Purkinje cell layer)

Having established that EAE caused cerebellar Purkinje cell injury, double immuno-labelling experiments for expression of calbindin-D_28K_ and EGFP were carried out to identify Purkinje cells that had fused with EGFP-expressing BM-derived cells in response to EAE and to characterise their phenotype. Such cells were scattered throughout the cerebellum of BM chimeric mice and indicated the expression of donated BM-derived nuclear transgenes (e.g. EGFP) within the host Purkinje cell (Fig. 3a). EGFP-positive Purkinje cells occurred at a mean frequency of 0.89% ± 0.41 (± SD, *n* = 10 animals), which, in agreement with previous work [20], was almost 20 times higher (*p* < 0.001; Mann–Whitney *U* test) than the incidence in control transplanted animals (0.05% ± 0.07, mean ± SD, *n* = 5 animals; transplanted with EGFP BM-derived cells but without induction of EAE).

**Fig. 4:**
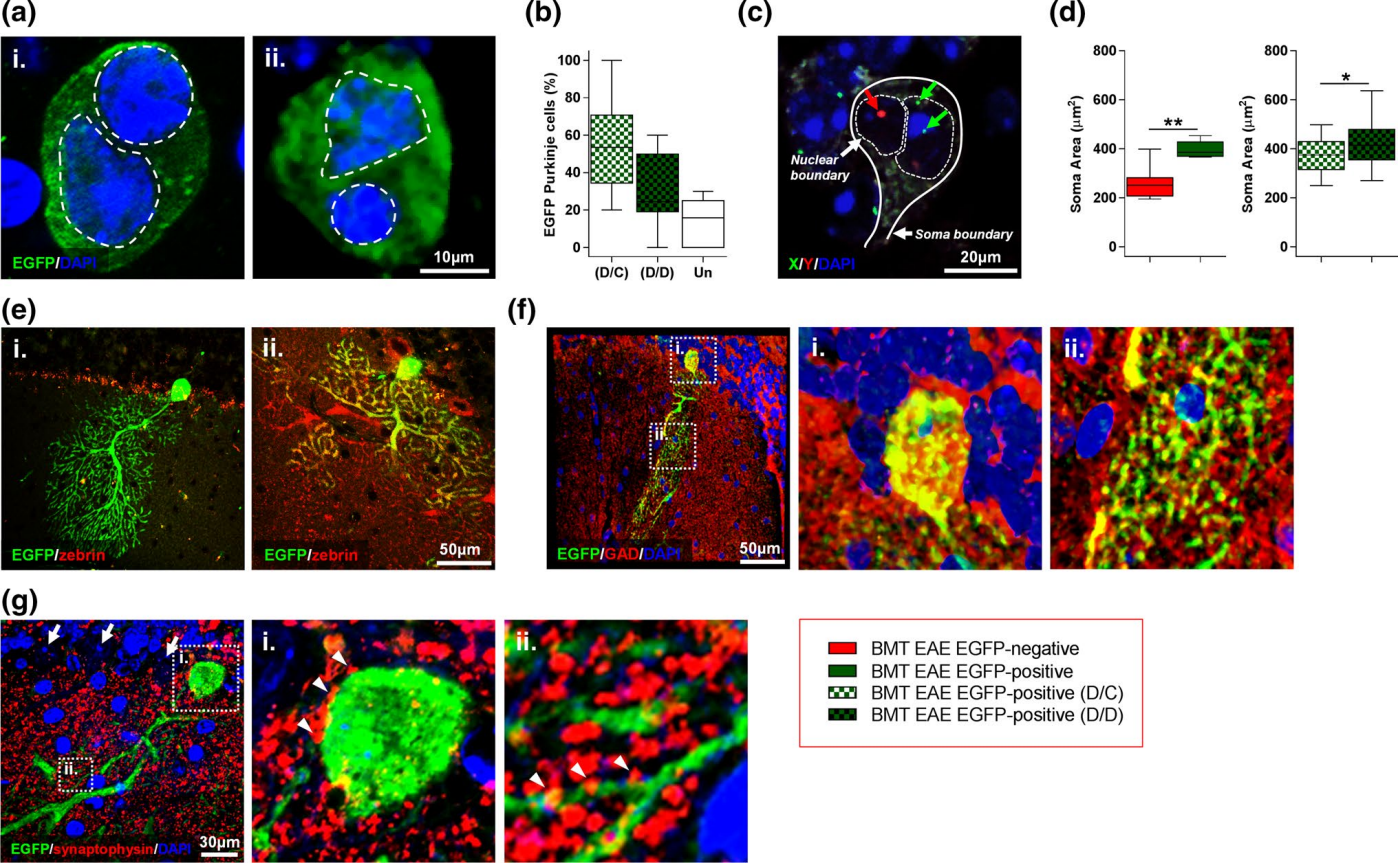
Phenotypic characteristics of EGFP-expressing Purkinje cells. **a** Images and **b** quantification of binucleate EGFP-positive Purkinje cells (Purkinje cells that had fused with EGFP-BM-derived cells) containing either (i) two Purkinje cell-like nuclei with dispersed chromatin (D/D, dispersed/dispersed) or (ii) one Purkinje cell-like nucleus and one smaller nucleus with compact chromatin (D/C, dispersed/compact). Un indicates nuclei that were unclassified in a minority of cells. **c** FISH analysis of male to female BM transplantation highlighting a binucleate Purkinje cell containing nuclei (dashed outlines) with both a female (X/X) and male (Y/?) karyotype, indicative of a fusion event between a male BM-derived cell and an endogenous female Purkinje cell. **d** Soma size of Purkinje cells in BM-transplanted mice with EAE (BMT EAE); (left) measurements shown are for EGFP-negative Purkinje cells (red), EGFP-positive Purkinje cells only (green), and (right) EGFP-positive Purkinje cells sorted according to density of nuclear chromatin (D/C, dispersed/compact; D/D, dispersed/dispersed). **e** Images of (i) a zebrin-negative, EGFP-positive Purkinje cell and (ii) a zebrin-positive, EGFP-positive Purkinje cell. **f** 3D laser scanning confocal images showing extensive GAD expression in the (i) soma and (ii) dendrites of an EGFP-positive Purkinje cell. **g** Evidence of synaptophysin-positive pre-synaptic boutons (white triangles) on the (i) soma and (ii) dendrites of an EGFP-positive Purkinje cell. (White arrows indicate EGFP-negative Purkinje cells). **p < 0.01 (**d**); Mann–Whitney *U* test. *p < 0.05 (**d**); unpaired *t* test. Box and whisker plots show median ± quartiles (box), min/max (whiskers). For all tests, *n* = ≥ 5 per group

The EGFP-positive Purkinje cells showed typical Purkinje cell morphology, characterised by extensive dendritic arborisation climbing through the molecular layer toward the surface of the cortex and an axon originating at the opposite (basal) pole of the cell soma (within the Purkinje cell layer) (Fig. 3b). In-depth confocal evaluation and serial reconstruction, by which it was possible to image the volume of the soma, revealed that EGFP-positive Purkinje cells were binucleate (Fig. 4a). The majority (61.5%) of these cells contained one large Purkinje cell-like nucleus with dispersed chromatin and a large nucleolus, and a second smaller nucleus with compact chromatin (Fig. 4b). This nuclear phenotype signifies a recent fusion event [41]. The remainder contained two Purkinje cell-like nuclei with dispersed chromatin, indicative of a more mature fused cell; the donor nucleus, identified by its compact chromatin, is thought to be reprogrammed to assume the properties of a Purkinje cell nucleus [41]. The different origin of each nucleus was verified with FISH using probes to both X and Y chromosomes. This showed that binucleate cells contained a complement of sex chromosomes indicative of fusion between a male and a female cell (XY + XX or Y + XX karyotype) (Fig. 4c). Furthermore, the soma of EGFP-positive Purkinje cells was significantly larger than that of adjacent or nearby EGFP-negative Purkinje cells (that had not undergone fusion) located in the same EAE animal, and the soma of fused Purkinje cells containing two dispersed nuclei was larger than that of fused cells with both a dispersed and a compact nucleus (Fig. 4d).

Further analysis revealed that hyper-phosphorylated neurofilament in Purkinje cells, detected by SMI-34 immunolabelling, was absent in all EGFP-positive Purkinje cells (*n* = 5 animals, ≥ 10 cells counted/per animal). This indicates that the increase in the number of SMI-34-positive Purkinje cells in response to EAE (Fig. 2a, c) reflects SMI-34 expression by non-fused, EGFP-negative Purkinje cells. Previous studies have shown differential degeneration of zebrin-positive (Z+) or zebrin-negative (Z−) Purkinje cells in mouse models of various cerebellar diseases [18, 36] but we found that equal proportions of EGFP-positive cells were either Z− (56.9% ± 33.51, mean ± SD, *n* = 6 animals) or Z+ (43.1% ± 33.51, mean ± SD, *n* = 6 animals) (*p* = 0.75; paired Wilcoxon matched-pairs signed rank test. ≥ 10 cells counted/per animal) (Fig. 4e), indicating that fusion with BM-derived cells did not favour either subtype of Purkinje cell. All fused cells examined also expressed the gamma-aminobutyric acid (GABA)-synthesizing enzyme GAD within the soma and the dendritic arbour, demonstrating maintained synthesis of the Purkinje cell neurotransmitter, GABA (Fig. 4f). Furthermore, labelling of synaptic terminals with synaptophysin showed that synaptic contacts onto the soma and dendrites were maintained from, respectively, basket cells and parallel fibres (Fig. 4g). Altogether, these results demonstrate that upon fusion with BM-derived cells, Purkinje cells are largely both structurally and phenotypically indistinguishable (with exception of binucleation) from normal ‘healthy’ Purkinje cells.

### Fusion between BM-derived cells and injured Purkinje cells preserves Purkinje cell firing properties

In parallel with the analysis of the structure and number of Purkinje cells in EAE mice that had or had not fused with BM-derived cells infiltrating the brain, we investigated how CNS inflammation and degeneration affects the firing properties of Purkinje cells and explored the possibility that fusion with BM-derived cells mitigates the effects of cell injury on electrical activity. Extracellular recordings of spontaneous firing were made from EGFP-positive and adjacent or nearby EGFP-negative Purkinje cells in the same cerebellar slices ex vivo, and their electrical activity was compared with that of Purkinje cells in slices from ‘healthy’ age and gender-matched control animals (female, 9.5–11.5 months, no BMT, no EAE-induction). For all three varieties of Purkinje cells, a minor proportion of cells did not fire during at least 10 min of recording. The percentage of silent EGFP-negative cells (34.6%; 9/26) was approximately double that of silent EGFP-positive cells (15.8%; 3/19) or silent control cells (19.4%; 6/31). However, these proportions were not statistically different [*χ*^2^(2, *n* = 76) = 2.695, *p* = 0.2598] possibly due to the relatively low fusion rate of less than 1% that, in turn, resulted in the infrequent occurrence of EGFP-positive cells near the surface of the slice that could be accessed with a recording electrode.

Electrically active Purkinje cells showed heterogeneity in their firing patterns, in keeping with the previously-described heterogeneity of Purkinje cell firing in control animals [15]. Therefore, to enable comparison of electrical activity, we firstly classified the firing patterns in cells from control animals into different categories and established the relative percentages of cells exhibiting the different patterns. The same criteria were then applied to recordings from EGFP-negative and EGFP-positive cells in slices from EAE animals. The three types of firing identified in control cells (see Online Resource 1; Fig. S1) were (1) tonic (characterised by the absence of long pauses), (2) trimodal (characterised by long pauses and periods of tonic and burst firing which could occur in any order) and (3) irregular (characterised by firing of single spikes or clusters of 2–4 closely spaced spikes, at widely varying intervals) (see “Materials and methods” for more analytical details).

**Fig. 5:**
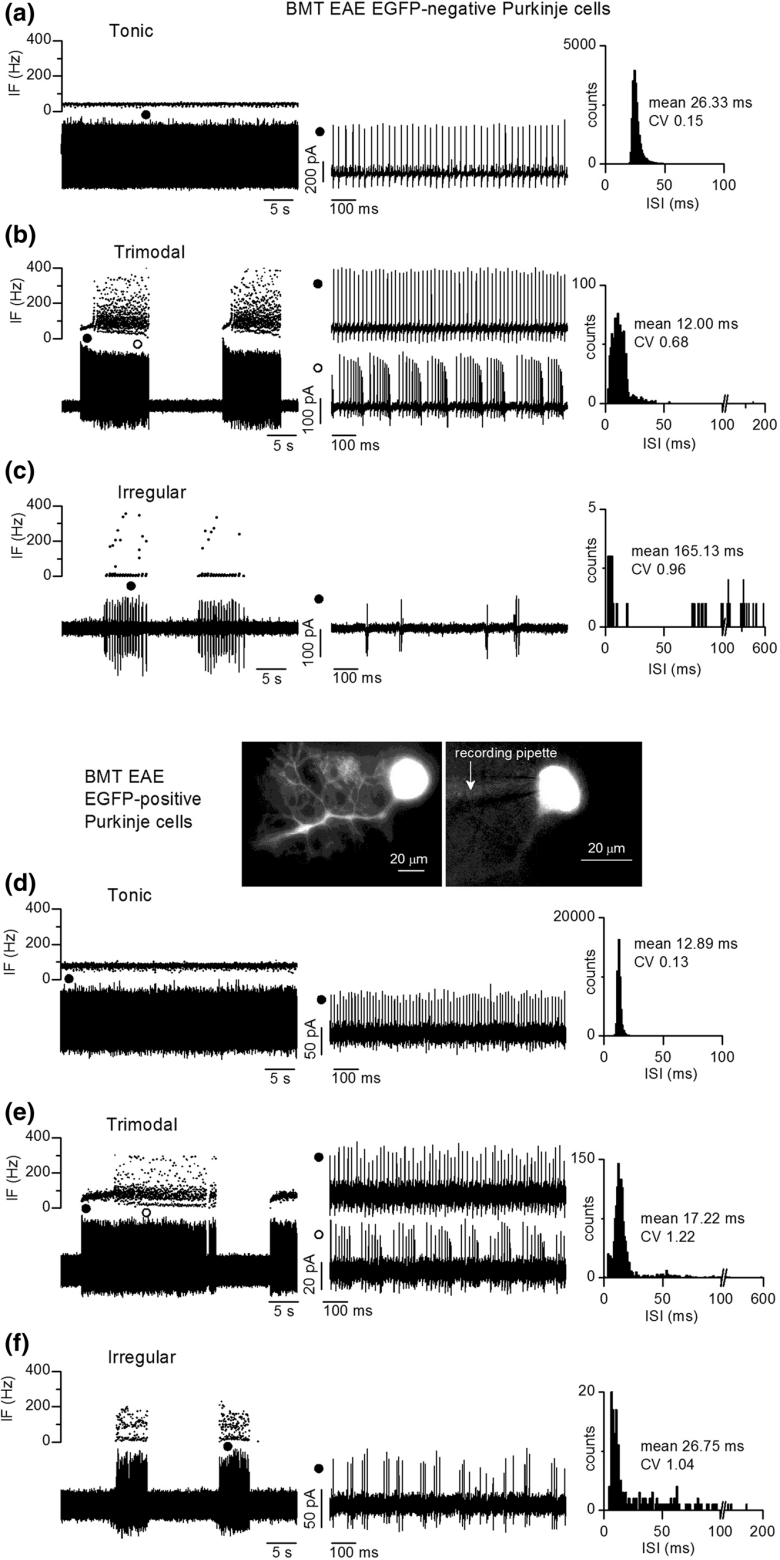
Heterogenous firing patterns of Purkinje cells that had or had not fused with BM-derived cells in EAE mice. **a**–**c** Different spontaneous firing patterns recorded from three Purkinje cells that had not fused with BM-derived cells, identified by lack of EGFP-expression. Extracellular recordings were made from Purkinje cells located in cerebellar slices ex vivo. Dissimilar patterns of firing were recognized according to criteria established for Purkinje cells from control animals (see Online Resource 1; Fig. S1). **a** Tonic firing in a regular pattern; IF frequency is constant (left upper) during a 40 s period of extracellular recording (left lower). The regular firing is visible in the expanded 1 s fragment of the current trace (middle, indicated by filled circle) and the narrow histogram (CV) of ISI with a single peak (right, the distribution was derived from 6 min of recording). **b** Trimodal firing; three firing modes (quiet, tonic and bursting) are evident in the plot of IF against time (left upper) and the continuous extracellular recording (left lower). Tonic and burst firing is evident in the expanded 1 s fragments of current traces (middle) and the multiple components and relatively high CV of the ISI histogram (right, generated for a single firing episode). **c** Irregular firing; in this cell, episodes of firing are separated by distinct quiet periods. IF fluctuates widely (left), the expanded current trace reveals the occurrence of spikes in clusters (middle) and the histogram of ISI during a firing episode (right) reveals extremely short and longer ISI within and between clusters of spikes. **d**–**f** Different spontaneous firing patterns recorded in three Purkinje cells that had fused with BM-derived cells, identified by EGFP-fluorescence. The images show a fluorescent cell identified with an epifluorescence microscope in an unfixed cerebellar slice (left) prior to recording from the soma with an extracellular recording pipette (right). Examples of cells firing **d** tonically, **e** trimodally and **f** irregularly, identified by the same criteria applied to EGFP-negative cells and cells from control animals

**Fig. 6:**
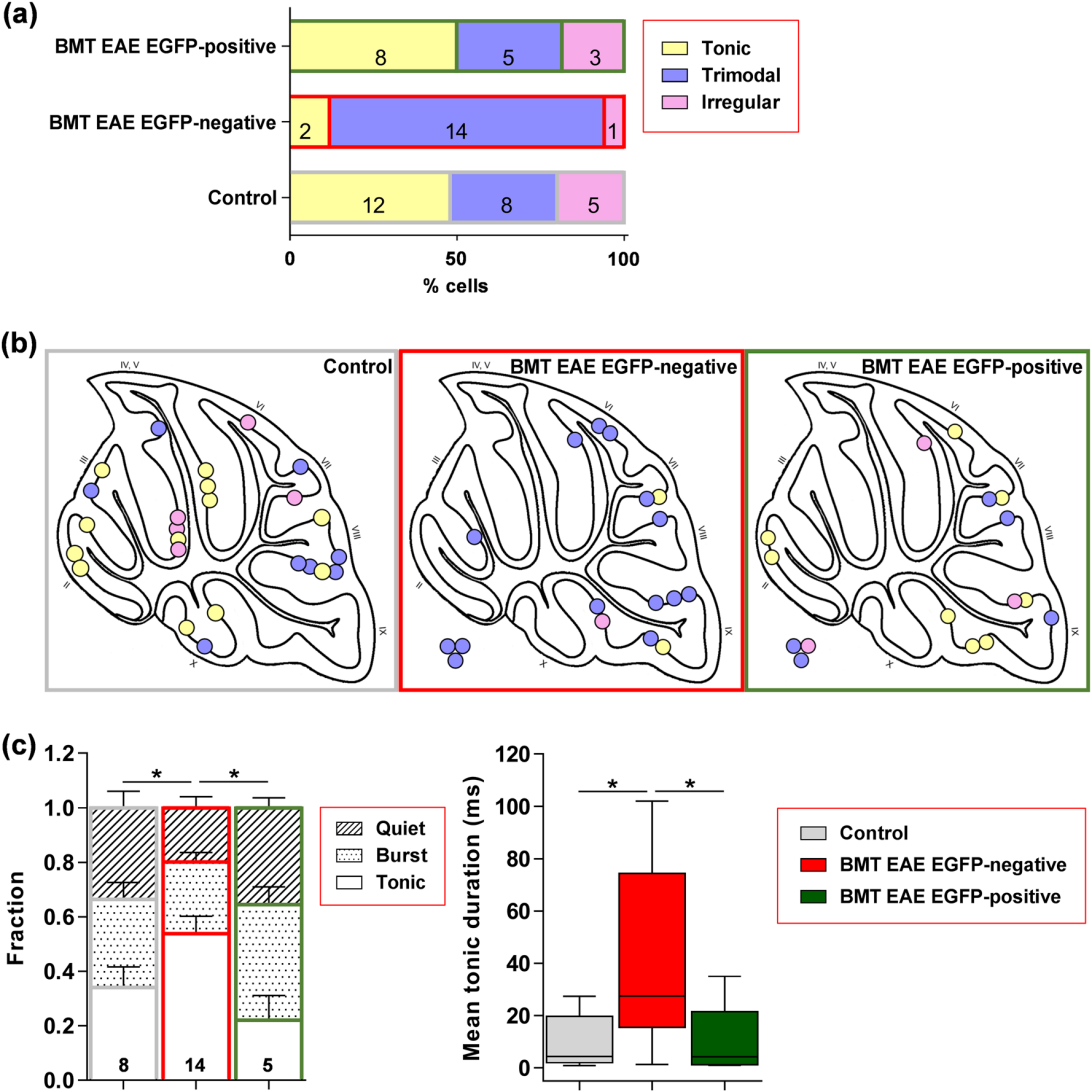
The proportions of Purkinje cells with different firing patterns are changed in EAE; this change is normalized by fusion with BM-derived cells. **a** Different percentages of cells with tonic, trimodal and irregular firing patterns recorded in EGFP-negative and EGFP-positive Purkinje cells (fused with BM-derived cells) in EAE-mice, and in Purkinje cells from control animals [*χ*^2^(4, *n* = 58) = 12.42, *p* = 0.0145]. **b** Locations of recorded cells, colour-coded according to the type of firing observed, on a schematic drawing of a sagittal slice of mouse cerebellum. For a few recordings (circles next to the drawing) there was no information about their location. Numbers indicate different lobules. **c** Left, fraction of time spent by trimodally firing cells in each of three modes, tonic, burst and quiet. EGFP-negative cells spend more time in the tonic mode than EGFP-positive cells or cells from control animals (*p < 0.05; 2-way ANOVA, Holm-Sidak’s multiple comparison test); Right, box plot of mean durations of tonic periods in trimodally firing cells. Tonic periods are longer in EGFP-negative cells (*p < 0.05; ANOVA followed by Holm-Sidak’s multiple comparison test). Box and whisker plots show median ± quartiles (box), min/max (whiskers). *n* values are depicted within graph bars

We found that the three types of firing present in Purkinje cells from control animals also occurred in EGFP-negative and EGFP-positive Purkinje cells of EAE animals, as illustrated in Fig. 5. However, the proportions of cells with the different patterns of firing were not the same. Within cerebellar slices from EAE animals, the majority of EGFP-negative cells (82%) fired in a trimodal pattern compared to only 31% of EGFP-positive cells (Fig. 6a); conversely, 50% of EGFP-positive cells fired tonically and this percentage was 4 times greater than for EGFP-negative cells (12%). These differences do not reflect different lobular locations as recordings were made from adjacent or nearby EGFP-positive and negative cells (Fig. 6b). Notably, the distribution of EGFP-positive cells with the different firing patterns was the same as for Purkinje cells from control animals (Fig. 6a), which were also recorded throughout different cerebellar lobules (Fig. 6b). Furthermore, the fraction of time spent by EGFP-negative, trimodal cells in the three different modes was not the same as for EGFP-positive or control trimodal cells. Overall, more time was spent in the tonic mode and the average tonic periods were longer in trimodal EGFP-negative cells (Fig. 6c). However, there were no differences in the frequency (mean ISI) or regularity of tonic firing (CV ISI) by tonic cells or trimodal cells (see Online Resource 1; Fig. S2), or in the minimum and maximum frequencies of burst firing by trimodal cells (not shown, *p* > 0.05 for all comparisons). These electrophysiological results demonstrate that a pathological environment causes an increase in the number of trimodally firing Purkinje cells and a decrease in the number of tonically firing Purkinje cells. This change can be prevented by fusion of BM-derived cells with Purkinje cells.

## Discussion

This study supports the hypothesis that by fusing with neurons in the adult brain, BM-derived cells can prevent or repair pathological changes triggered in mature brain neurons by neuroinflammation. We show that the cell fusion in vivo maintains not only the structure and subcellular composition of cerebellar Purkinje cells; it also maintains the electrical firing properties of these neurons. Our study finally establishes a likely functional role for heterotypic cell fusion, a process identified more than a decade ago [2, 41] and proposed as a mechanism for the existence of Purkinje cells with two nuclei, first recognised almost 80 years ago [3]. The conservation of normal spontaneous firing typical of the original native neuron demonstrates that the heterokaryon retains neuronal function, which is to fire action potentials and hence transfer information through the neuronal circuitry.

Neuroinflammation induced by EAE caused both structural and functional abnormalities in cerebellar Purkinje cells. There was also an increased incidence (20-fold) of fusion between BM-derived cells and Purkinje cells; these elevations in fusion are similar to those reported in EAE mice by Johansson et al. [20]. Pathologically, we found demyelination of Purkinje cell axons in the cerebellar cortex and evidence of injury to both the Purkinje cell axon and soma; these changes bear significant resemblance to features of multiple sclerosis, where abnormal neurofilament phosphorylation in Purkinje cells and robust Purkinje cell loss within lesional areas are reported [5, 11, 25, 34]. The shift from a tonic mode to a trimodal mode of firing of Purkinje cells in ex vivo cerebellar slices represents an increased tendency to burst rather than tonic firing. This is in agreement with the altered firing patterns recorded previously from in vivo Purkinje cells in EAE animals [35]. Aberrant expression of the sensory neuron specific sodium channel, Na_v_1.8, may be partly responsible [35] although changes in expression levels of the various ion channels and transporters that shape Purkinje cell firing are likely to be implicated [5, 7, 11, 16, 17]. It is possible, therefore, that fusion with BM-derived cells maintains Purkinje cell firing and/or interaction with neighbouring cells through normalisation of ion channel, transporter or receptor expression. This is predicted to occur by nuclear transfer from the BM-derived cells and subsequent exposure to transcription factors within the Purkinje cell fusion partner, resulting in the expression of donated genes to restore ‘normal’ gene expression. A clue that this occurs is the observed translation of the *EGFP* gene, donated by the BM-derived cell following fusion. Further insight into the genes expressed by fused Purkinje cells could be obtained through exploiting species-mismatched BMT (transplantation of rat BM into mice [20]). Subsequent single-cell mRNA analysis of fused cells, using species-specific primers to genes involved in neurophysiological function known to be differentially expressed in EAE (e.g. *Cacna1a*, *Scn10a*; *S100a10* [5, 7, 11]), may provide key answers. Characterisation of the corrective changes in gene expression and identification of the channel or transporter genes involved requires further study.

In response to cerebellar injury, why BM-derived cells fuse preferentially with Purkinje cells is unknown. Purkinje cells are likely to express membrane-specific molecules rendering them fusogenic. As there is no evidence for Purkinje cell generation in adult life [28], the potential evolutionary-driven capacity to express these molecules, allowing the targeted fusion of BM cells with Purkinje cells to prevent their loss, could relate to their unique physiological significance in the cerebellum. The notion that cell fusion in the brain may have a role in neuronal repair has significant implications for regenerative medicine. Degeneration of the cerebellum, and particularly Purkinje cells, occurs in numerous acquired and inherited neurological disorders [4, 6, 19, 24, 34, 37]. Moreover, many of these disorders are thought to share common pathogenic pathways that result in altered intrinsic activity of Purkinje cells [32].

BM-derived cells fusing with Purkinje cells in response to inflammation is of particular importance to cerebellar injury, as cell fusion may be an immune-mediated phenomenon aimed at protecting Purkinje cells against inflammatory or toxic insults [21]. As we report here in mice, inflammatory disease-associated increases in BM-derived cells fusing with Purkinje cells have also been observed in patients who had multiple sclerosis [22]. There is also evidence that underlying levels of cerebellar inflammation influence Purkinje cell fusion and heterokaryon formation in patients with genetic ataxias [24]. Exploiting heterotypic cell fusion as a mode of cell rescue, to introduce ‘therapeutic’ genetic material to boost neuronal cell survival may, therefore, hold hugely valuable implications for a wide range of patients with otherwise untreatable neurodegenerative or inflammatory disease. Indeed, there are equally important implications for aging in the CNS and the preservation of highly complex neuronal sub-populations.

Whole BM and selected BM-derived haematopoietic and mesenchymal stem cell fractions have been studied experimentally with the ultimate purpose of treating cerebellar disease [12]. Yet, there are still significant deficiencies in our understanding of which precise BM cell therapy protocol is most efficacious. Central to this, understanding whether selected BM cell populations or the whole BM are required to reduce disease activity and effect repair is of paramount importance. Understanding whether there are fundamental differences in the ‘fusogenic’ properties of different BM cell types will help aid the design of future therapeutic trials utilizing transplantation of BM cells in these disorders. Exploring these precise interactions between BM-derived cells and Purkinje cells is likely to aid the search for much needed novel reparative and regenerative therapeutic approaches into ways in which nerve cells can be protected and their survival prolonged.

## Electronic supplementary material

Below is the link to the electronic supplementary material.

Supplementary material 1 (PDF 258 kb)
