# Supplementary material for: Aberrant cerebellar Purkinje cell function repaired in vivo by fusion with infiltrating bone marrow-derived cells
Source: Acta Neuropathol. 2018 Mar 14;135(6):907–21. doi: 10.1007/s00401-018-1833-z (PMC5954067; doi:10.1007/s00401-018-1833-z)
Supplement: Supplementary file 1 — Supplementary material 1 (PDF 258 kb) [file 401_2018_1833_MOESM1_ESM.pdf]

Acta Neuropathologica

Supplementary Material.

Aberrant cerebellar Purkinje cell function repaired in vivo by fusion with infiltrating bone marrow-derived cells

Kevin C. Kemp [1], Rimi Dey [1], Johan Verhagen [3‡], Neil J. Scolding [1], Maria M. Usowicz [2†] and Alastair Wilkins [1†]*

[1.] *Multiple Sclerosis and Stem Cell Group, Translational Health Sciences, Bristol Medical School, University of Bristol, Bristol, UK.*

[2.] *School of Physiology, Pharmacology and Neuroscience, University of Bristol, Bristol, UK.*

[3.] *Infection and Immunity, School of Cellular and Molecular Medicine, University of Bristol, Bristol, UK.*

[†] ***These authors contributed equally***

[‡] ***Current address****: - Department of Immunobiology, Faculty of Life Sciences & Medicine, King's College London, Guy's Hospital, London, UK.*

Corresponding author

***** Correspondence: e-mail: kevin.kemp@bristol.ac.uk

Supplementary Figures and Legends

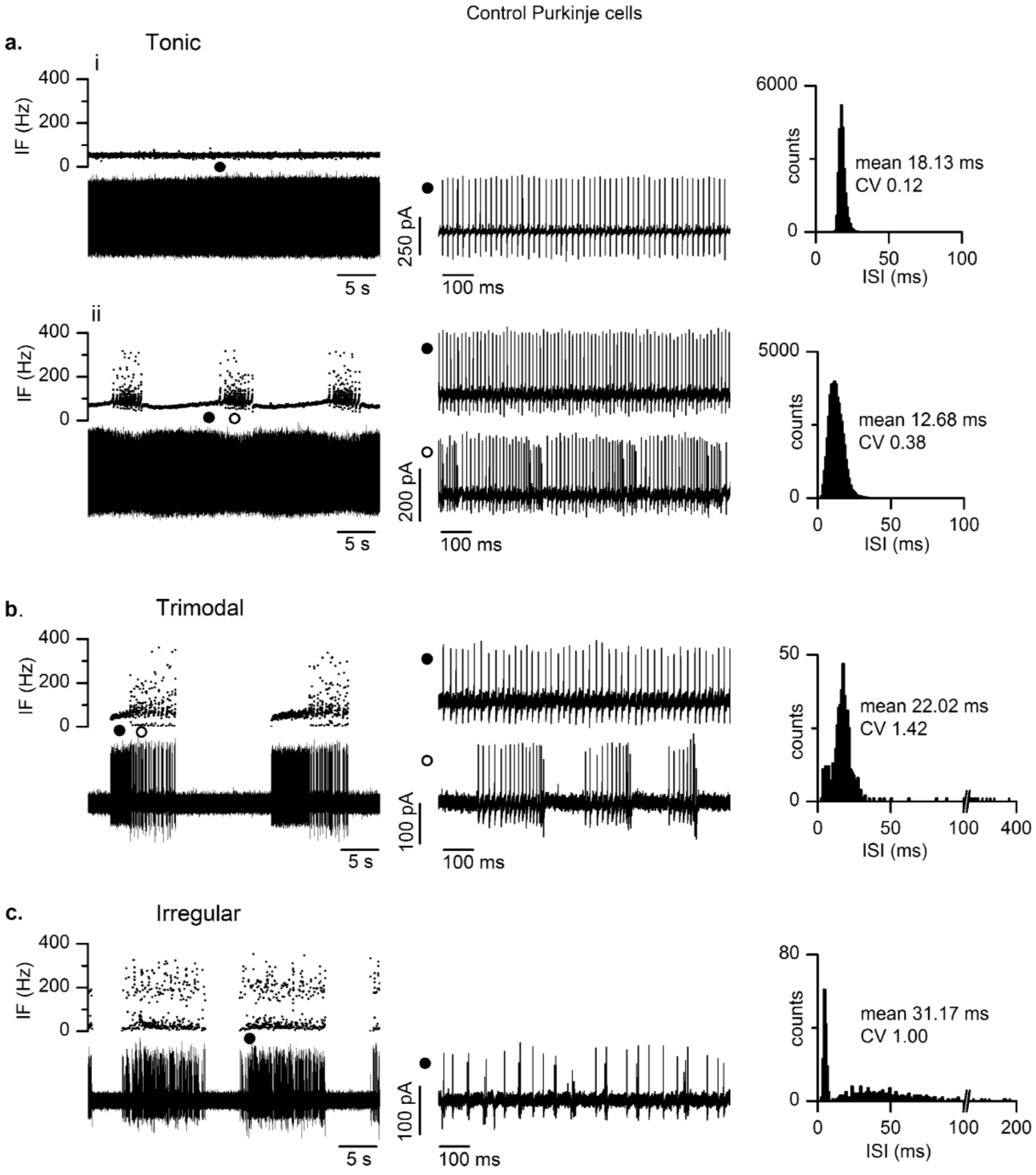

Fig. S1 Heterogeneous firing patterns of Purkinje cells in cerebellar slices from adult control C57BL/6 mice (female, aged 9.5 – 11.5 months). **(a)** Examples of tonic spontaneous electrical activity. *Left*, plot of instantaneous firing (IF) frequency against time (*upper*) during a 40 s period of extracellular recording (*lower*). **(a.i)** IF is constant, denoting regular tonic firing; (**a.ii**) periods of constant IF are interspersed with sharp increases in IF, denoting alternate regular

and burst firing. *Middle*, expanded 1 s fragments of current traces on the left, at times indicated by symbols (filled or empty circles). *Right*, histograms of interspike intervals (ISI) for recordings from cells depicted on the left. Each distribution, derived from 7 minutes of recording, shows a single peak and is described by the mean and CV indicated. All ISI were shorter than 40 ms. **(b)** Example of spontaneous activity with a trimodal pattern of firing. *Left*, three firing modes, quiet, tonic and bursting, are evident from the plot of IF against time (*upper*) and the continuous extracellular recording (*lower*). *Middle*, expanded 1 s fragments of current traces during a firing episode (times indicated by symbols), illustrating tonic and burst firing. *Right*, histogram of ISI during a firing episode. The ISI distribution has multiple components and a higher CV and bigger values (up to 400 ms) than in **(a)** because of distinct ISI between spikes during regular firing, and within and between bursts. **(c)** Example of irregular spontaneous activity. In this cell (but not in all cells placed in this category) firing episodes are separated by distinct quiet periods. *Left*, IF fluctuates between 4 - 90 Hz and 120 – 350 Hz. *Middle*, an expanded 1 s fragment of the current trace on the left reveals the occurrence of spikes as individual events or in clusters of two, three or four events. *Right*, histogram of ISI during a firing episode. The short component (< 9 ms) depicts intervals within clusters of spikes.

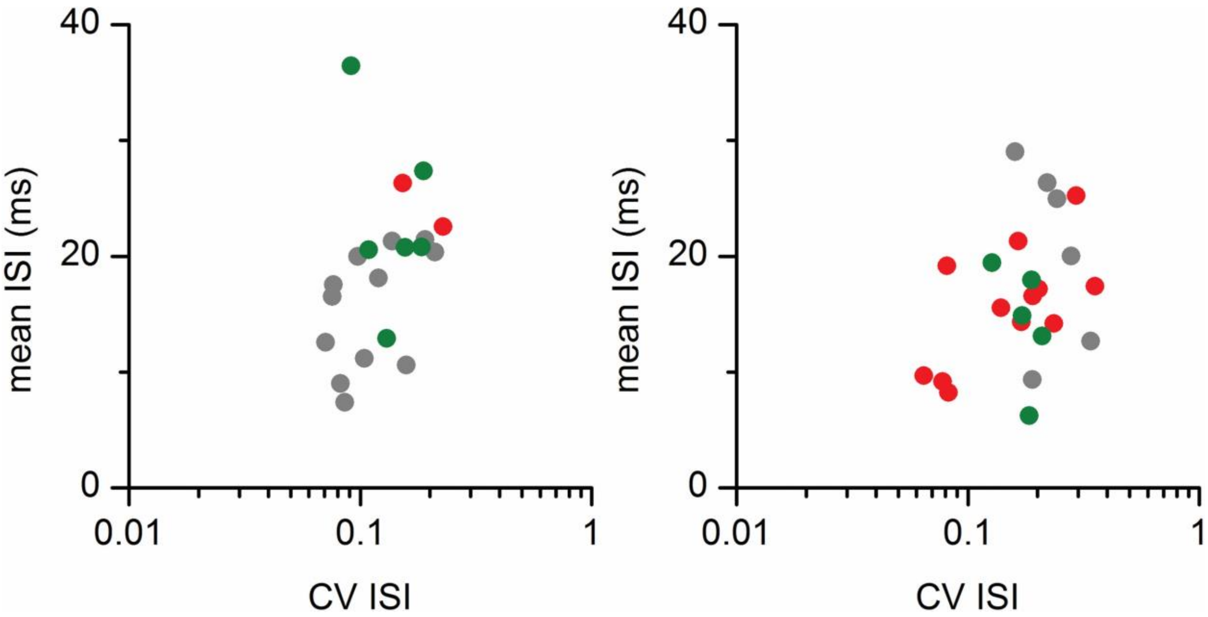

Fig. S2 Regularity and frequency of tonic firing do not differ between EGFP-negative and positive Purkinje cells of BMT EAE-animals and Purkinje cells of control animals. Scatter plots of mean ISI against CV ISI for cells firing in isolated cerebellar slices with a regular tonic pattern (*left*) and for tonic periods of firing in cells firing trimodally (*right*). *Red symbols*, EGFP-negative cells from BMT EAE animals; *green symbols*, EGFP-positive cells from BMT EAE animals; *grey symbols*, control animals.
